# Combining transcriptomics and metabolomics to reveal the underlying molecular mechanism of ergosterol biosynthesis during the fruiting process of *Flammulina velutipes*

**DOI:** 10.1186/s12864-019-6370-1

**Published:** 2019-12-19

**Authors:** Ruihong Wang, Pengda Ma, Chen Li, Lingang Xiao, Zongsuo Liang, Juane Dong

**Affiliations:** 1 College of Life Sciences, Northwest A&F University, Yangling, 712100 China; 2 Shaanxi Zhongxing Gaoke Biological Technology Co., Ltd, Yangling, 712100 China; 3 College of Life Sciences, Zhejiang Sci-Tech University, Hangzhou, China

**Keywords:** *Flammulina velutipes*, Transcriptomics, Metabolomics, Combined analysis, Ergosterol biosynthesis, Fruiting process

## Abstract

**Background:**

*Flammulina velutipes* has been recognized as a useful basidiomycete with nutritional and medicinal values. Ergosterol, one of the main sterols of *F. velutipes* is an important precursor of novel anticancer and anti-HIV drugs. Therefore, many studies have focused on the biosynthesis of ergosterol and have attempted to upregulate its content in multiple organisms. Great progress has been made in understanding the regulation of ergosterol biosynthesis in *Saccharomyces cerevisiae*. However, this molecular mechanism in *F. velutipes* remains largely uncharacterized.

**Results:**

In this study, nine cDNA libraries, prepared from mycelia, young fruiting bodies and mature fruiting bodies of *F. velutipes* (three replicate sets for each stage)*,* were sequenced using the Illumina HiSeq™ 4000 platform, resulting in at least 6.63 Gb of clean reads from each library. We studied the changes in genes and metabolites in the ergosterol biosynthesis pathway of *F. velutipes* during the development of fruiting bodies. A total of 13 genes (6 upregulated and 7 downregulated) were differentially expressed during the development from mycelia to young fruiting bodies (T1), while only 1 gene (1 downregulated) was differentially expressed during the development from young fruiting bodies to mature fruiting bodies (T2). A total of 7 metabolites (3 increased and 4 reduced) were found to have changed in content during T1, and 4 metabolites (4 increased) were found to be different during T2. A conjoint analysis of the genome-wide connection network revealed that the metabolites that were more likely to be regulated were primarily in the post-squalene pathway.

**Conclusions:**

This study provides useful information for understanding the regulation of ergosterol biosynthesis and the regulatory relationship between metabolites and genes in the ergosterol biosynthesis pathway during the development of fruiting bodies in *F. velutipes*.

## Background

Edible fungi are the sixth largest crop in China with a total output of 33 million tons in 2015 [1]*. Flammulina velutipes* (*F. velutipes*) has been recognized as a model industrial basidiomycete; it is one of the most commonly used edible fungi, serving as an excellent source of vitamins, amino acids, polysaccharides, fibre, terpenoids, phenolic acids, steroids, fatty acids and other metabolites, and is widely cultivated worldwide [2–5]. Compounds with pharmaceutical value can be isolated from the fruiting bodies or mycelia of *F. velutipes*, including anti-inflammatory and immunomodulatory proteins [6], antitumour, antioxidant and acetylcholinesterase inhibitory polysaccharides, antitumour agglutinins and immunomodulatory compounds [7], antimicrobial terpenoids [8], and antitumour and antioxidant sterols [9, 10]. The active antitumour sterols include ergosterol, 22,23-dihydroergosterol, ergosta-5,8,22-trien-3-ol and ergo-8(14)-ene-3-ol [11, 12]. The chemical composition of sterols is mainly ergosterol (54.8%) and 22,23-dihydroergosterol (27.9%) [10]. GC-MS or HPLC studies of saponification extraction have revealed that the ergosterol content in *F. velutipes* was 35.5 mg/100 g in wet weight or 68.0 mg/100 g in dry weight [13, 14].

Ergosterol (C_28_H_43_OH) is a typical fugal sterol and an important constituent of various membrane structures of fungal cells, and it contributes to multiple physiological functions in cells, such as cell viability, membrane permeability, membrane fluidity, membrane integrity and intracellular transport. Therefore, when ergosterol is lacking, abnormal cell membrane function and even cell rupture may occur [15]. In recent years, a variety of fungicides collectively known as sterol biosynthesis inhibitors (SBIs) have been successfully developed to target certain enzymes or end products of the ergosterol biosynthesis pathway and have been widely used in medicine and agricultural production [16]. More importantly, ergosterol and some of its biosynthetic intermediates have great economic value. In the pharmaceutical industry, ergosterol is an important precursor of vitamin D2, progesterone, hydrocortisone, and brassinolide, and the products of almost all steps of its biosynthesis are drug precursors [17, 18].

Ergosterol and its derivatives are obtained mainly by chemical synthesis, genetic engineering and metabolic engineering [19]. Because of the various steps, long route, low efficiency and high cost involved, chemical synthesis of ergosterol and its derivatives is not the preferable way to obtain these compounds. One of the main approaches for producing ergosterol and its derivatives includes metabolic engineering of yeast, but because the content of ergosterol in cells is low, this production method is not efficient [20]. The biosynthesis of ergosterol is an extremely complicated process. Transcriptional regulation of the expression of related genes is one of the main means of adjusting ergosterol biosynthesis, and feedback regulation can play an important role in ergosterol production [21, 22]. Sterol regulatory element*-*binding proteins (SREBPs) are transcription factors that bind to the sterol regulatory element DNA *s*equence. Therefore, the manipulation of biosynthesis genes by genetic engineering may be an effective way to modulate sterol biosynthesis and intracellular sterol components. Although progress has been made in the metabolic and genetic engineering of synthetic pathways in *Saccharomyces cerevisiae* (*S. cerevisiae*), the roles of ergosterol biosynthesis genes in fruiting body growth and associated metabolic changes remain a mystery.

**Fig. 1:**
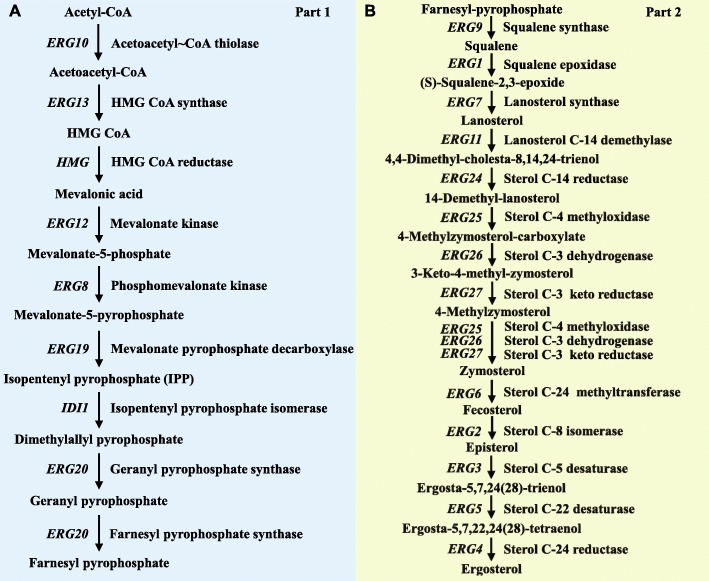
The biosynthesis pathway of ergosterol in *S. cerevisiae*. Biosynthesis intermediates, end products, and enzymes involved in ergosterol biosynthesis are indicated. **a** The mevalonate pathway is the first part, indicated in blue. **b** The post-squalene pathway is the second part, indicated in yellow. Enzyme names are shown next to each step. This figure was modified from Hu *et al*. [23]

The ergosterol biosynthetic pathway can be divided into two parts: the mevalonate pathway and the post-squalene pathway (Fig. 1). Part 1 includes nine steps (Fig. 1a) in the synthesis of farnesyl pyrophosphate from acetyl-CoA. The first step produces acetoacetyl-CoA from two acetyl-CoA molecules whose formation was previously catalysed by acetoacetyl-CoA thiolase (ERG10) [23]. Then, ERG13, HMG, ERG12, ERG8, ERG19, IDI1 and ERG20 successively catalyse eight reactions to produce farnesyl pyrophosphate from acetoacetyl-CoA. The enzymes in the mevalonate pathway are essential genes that are conserved in eukaryotes [24, 25]. Part 2 comprises 14 steps in the production of ergosterol from farnesyl pyrophosphate (Fig. 1b). The first step forms squalene from farnesyl pyrophosphate, and the squalene is then converted into lanosterol by squalene cyclization. Ergosterol is derived from lanosterol through steps regulated or catalysed by *ERG7*, *ERG11*, *ERG24*, *ERG25*, *ERG26*, *ERG27*, *ERG6*, *ERG2*, *ERG3*, *ERG5* and *ERG4* [26]. As ergosterol biosynthesis is regulated by both biosynthesis regulatory genes and environmental factors, genetic engineering and the optimization of culture conditions are the two main methods for increasing ergosterol productivity. For example, oxidative-fermentative growth combined with ethanol stimulation can increase ergosterol productivity [27]. Thus, the regulation of ergosterol biosynthesis is a complex process involving multiple factors.

Multi-omics has become a common biological approach for systematic genome analyses [28, 29]. In this study, we studied the first transcriptome and metabolome of *F. velutipes* samples from three developmental stages: the mycelia stage (FrI), the young fruiting bodies stage (FrII) and the mature fruiting bodies stage (FrIII). The transcriptome technique was used to identify changes in the expression of genes involved in the ergosterol biosynthesis pathway during fruiting body development. Thereafter, the metabolites in this pathway were completely scanned by nontargeted metabolomic techniques. We explored the regulatory relationship between genes and ergosterol biosynthesis during fruiting body development. The results had vital significance for understanding the metabolic pathway of ergosterol biosynthesis in *F. velutipes*.

## Results

### The analysis of RNA-Seq data

**Fig. 2:**
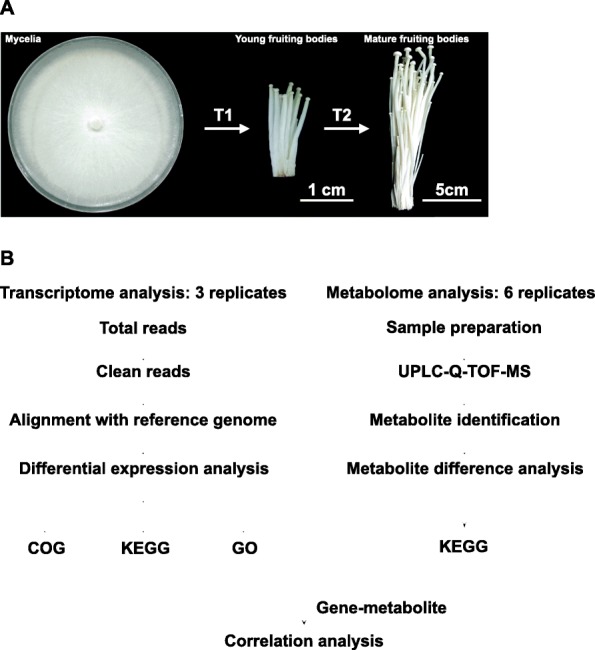
Pipelines of transcriptome and metabolome analysis of *F. velutipes*. **a** Mycelia, young fruiting bodies and mature fruiting bodies of *F. velutipes*. The scale bar of each figure is shown in the lower right corner. **b** Analysis pipelines of the transcriptome and metabolome of *F. velutipes*

In this study, nine libraries (FrI_1, FrI_2, FrI_3, FrII_1, FrII_2, FrII_3, FrIII_1, FrIII_2 and FrIII_3) from *F. velutipes* at three different developmental stages were prepared and sequenced using the Illumina HiSeq™ 4000 platform (Fig. 2). An overview of sequencing is given in Additional file 1: Table S1. After data filtering, approximately 60.29 Gb of clean reads was obtained, and at least 6.63 Gb of clean reads was generated for every library. The Q30 of each sample was approximately 92%, suggesting that the sequence data were accurate. These results demonstrated that the transcriptional profiling datasets presented satisfactory reliability for further analysis. After data filtering, the clean reads were aligned to the reference genome, and the statistical results are shown in Table 1. The ratio of mapped reads to the reference genome was approximately 82.0%.

**Table 1:** Mapping results of *F. velutipes* transcriptome

Sample	All Reads Num	Mapped Reads	Unmapped Reads
FrI_1	6983516	5704136 (81.68%)	1279380 (18.32%)
FrI_2	7004487	5714961 (81.59%)	1289526 (18.41%)
FrI_3	7088373	5783404 (81.59%)	1304969 (18.41%)
FrII_1	7046430	5752001 (81.63%)	1294429 (18.37%)
FrII_2	6994001	5711301 (81.66%)	1282700 (18.34%)
FrII_3	6994001	5727387 (81.89%)	1266614 (18.11%)
FrIII_1	6983516	5761401 (82.50%)	1222115 (17.50%)
FrIII_2	7004487	5763992 (82.29%)	1240495 (17.71%)
FrIII_3	7088373	5853578 (82.58%)	1234795 (17.42%)

### Functional annotation and pathway enrichment of differentially expressed genes (DEGs)

**Fig. 3:**
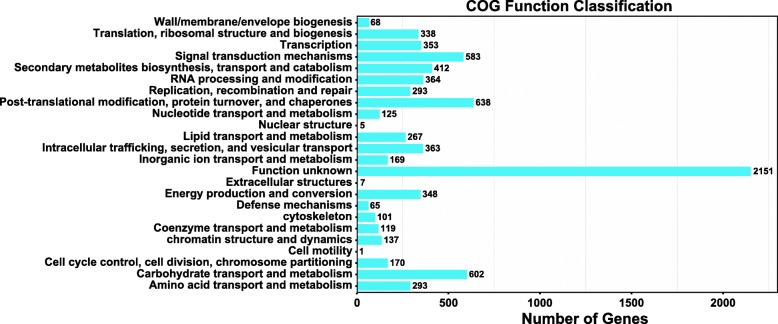
COG categories of the differentially expressed genes in *F. velutipes*

A total of 4907 (2798 downregulated and 2109 upregulated) DEGs were identified during the first developmental transition (T1), and 1383 (551 downregulated and 832 upregulated) DEGs were identified during the second developmental transition (T2) (Additional file 1: Figure S1). COG assignments were used to predict and classify the possible functions of the unique sequences, and describe gene evolution processes. In this study, COG annotation functions and the COG-annotated putative proteins were classified into 24 functional groups. As shown in Fig. 3, 26.83% (2151) of the DEGs did not have COG or belonged to the category with unknown function. In total, 7.96% of the DEGs were annotated with post-translational modification, protein turnover, and chaperones; 7.51% were annotated with carbohydrate transport and metabolism; 7.27% were annotated with signal transduction mechanisms and 5.14% were annotated with secondary metabolite biosynthesis, transport and catabolism.

The GO functional annotation and classification of DEGs during T1 and T2 were assigned 45 and 42 significant shared terms, respectively, which are displayed in Additional file 1: Figure S2 and S3. The results showed that the metabolic process, cellular process, single-organism process, localization, biological regulation, cellular component organization or biogenesis and regulation of biological process terms were significantly shared GO terms in the biological process category. Membrane, cell, cell part, organelle, membrane part, macromolecular complex and organelle part were the most shared terms in the cellular component category. Catalytic activity, binding, transporter activity and structural molecule activity were markedly shared terms in the molecular function category.

**Fig. 4:**
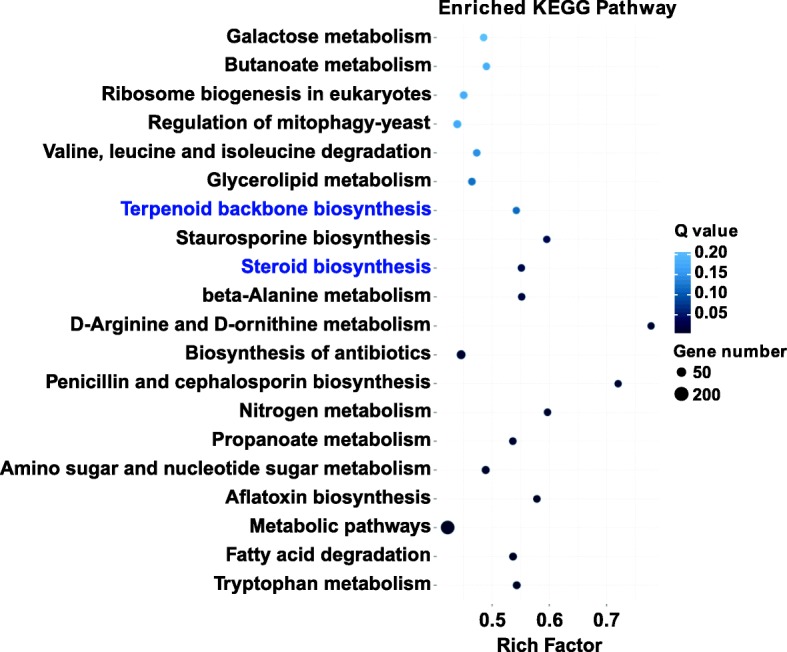
KEGG enrichment showing the top 20 metabolic pathways involving the DEGs during T1 of *F. velutipes*

KEGG pathway analysis revealed that diverse pathways were represented in the transcriptome dataset, with 5444 DEGs assigned to 121 pathways. From the bubble map of the DEG pathway enrichment analysis (only the top 20 metabolic pathways are shown) (Fig. 4), we found that two metabolic pathways related to ergosterol biosynthesis with significant enrichment were the terpenoid backbone biosynthesis pathway (ko00900) and the steroid biosynthesis pathway (ko00100).

### DEGs related to ergosterol biosynthesis

We analysed the essential genes involved in the terpenoid backbone biosynthesis pathway and the steroid biosynthesis pathway in *F. velutipes*. The results, revealed in Table 2 show that 13 genes (6 upregulated and 7 downregulated) were differentially expressed during T1. In addition, only 1 gene (1 downregulated) in the two metabolic pathways was differentially expressed during T2. It was found that the DEGs involved in the ergosterol biosynthesis process were concentrated mainly in T1. To validate the reliability of the transcriptome data, the sequences of 12 DEGs were analysed with RT-qPCR primers. The results of the RT-qPCR analysis exhibited close similarity to the RNA-Seq results, as shown in Additional file 1: Figure S4.

**Table 2:** The DEGs related to ergosterol biosynthesis at three different developmental stages of *F. velutipes*

Pathway	Gene_name	Gene_id	Ko id	EC no.	Regulation T1	Regulation T2
MVA pathway	ERG10	chromosome11:Gene1003	K00626	2.3.1.9	Down	NS
	ERG8	chromosome7:Gene953	K00938	2.7.4.2	Up	NS
	ERG19	chromosome7:Gene204	K01579	4.1.1.33	Down	NS
	IDI1	chromosome8:Gene1189	K01823	5.3.3.2	Down	NS
Post-squalene pathway	ERG9	chromosome9:Gene1056	K00801	2.5.1.21	Up	NS
	ERG1	chromosome5:Gene746	K00511	1.14.14.17	Down	NS
	ERG1	chromosome9:Gene102	K00511	1.14.14.17	Up	NS
	ERG7	chromosome6:Gene601	K01852	5.4.99.7	Up	NS
	ERG25	chromosome3:Gene262	K07750	1.14.13.72	Down	Down
	ERG25	chromosome10:Gene1780	K07750	1.14.13.72	Down	NS
	ERG26	chromosome5:Gene519	K07748	1.1.1.170	Up	NS
	ERG27	chromosome9:Gene636	K09827	1.1.1.270	Up	NS
	ERG3	chromosome1:Gene281	K00227	1.14.19.20	Down	NS

### Metabolic differences among the three different developmental stages of *F. velutipes*

**Fig. 5:**
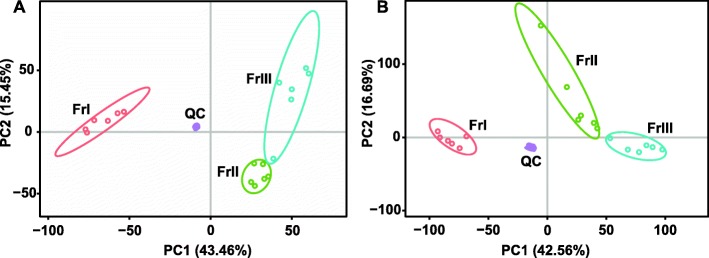
PCA and differential expression analysis of the *F. velutipes* metabolomes of different developmental groups. **a** PCA of positive ions. **b** PCA of negative ions

RNA-Seq analysis results indicated significant differences in metabolism during the development of *F. velutipes*; therefore, we investigated the changes in metabolic constituents over the three developmental stages. In this study, we used 18 samples (three stages × 6 biological replicates) to observe differences in metabolic constituents among the three developmental stages of *F. velutipes*. The metabolome used the VIP values of the first two principal components of the multivariate PLS-DA model and a combined univariate analysis of fold change and *p*-value to screen for differentially expressed metabolites. The screening conditions are as follows: 1) VIP ≥ 1; 2) fold change ≥1.2 or ≤ 0.83 and 3) *p*-value < 0.05. These three factors were taken into account to obtain a common ion. Metabolic pathway analysis was based on the KEGG database. To compare the metabolic constituents in the three developmental stages, datasets obtained from UPLC-TOF-MS in the ESI^+^ (ESI^−^) mode were subjected to PCA. The results showed different metabolic profiles among the three groups (Fig. 5). Indeed, the first principal component (PC2) in ESI^+^ mode (15.45% of the total variables) and PC1 in ESI^−^ (42.56%) were clearly separated between the FrI and FrII groups. The differences between the FrII and FrIII groups resulted from PC2 (15.45% variables) in ESI^+^ mode and PC2 (16.69%) in ESI^−^ mode. A total of 1742 (2154) and 751 (944) mass ions were selected between the FrI and FrII groups and between the FrII and FrIII groups in the ESI^+^ (ESI^−^) mode, respectively (Additional file 1: Table S2).

### Different accumulation of sterol derivatives at three developmental stages of *F. velutipes*

To understand the metabolic changes in ergosterol biosynthesis, we compared the metabolic profiles of *F. velutipes* at different developmental stages (Tables 3 and 4). In this study, we identified 17 metabolites involved in ergosterol biosynthesis, namely, mevalonate, mevolonate-5-phosphate, isopentenyl pyrophosphate, dimethylallyl pyrophosphate, farnesyl pyrophosphate, squalene-2-3-epoxide, lanosterol, 4,4-dimethyl-cholesta-8,14,24-trienol, 14-demethyl lanosterol, 4-methylzymosterol-carboxylate, 3-keto-4-methylzymosterol, 4-methylzymosterol, fecosterol, episterol, ergosta-5,7,24(28)-trienol, ergosta-5,7,22,24(28)-tetraenol and ergosterol, which are listed in Additional file 1: Table S3 and S4. Seven of the 17 metabolites exhibited significantly different expression levels during T1 (Table 3). Among these metabolites, the expression levels of three metabolites (isopentenyl pyrophosphate, dimethylallyl pyrophosphate and 4-methylzymosterol) were significantly increased, and the expression levels of 4 metabolites (ergosta-5,7,22,24(28)-tetraenol, 4,4-dimethyl-cholesta-8,14,24-trienol, 4-methylzymosterol-carboxylate and squalene-2-3-epoxide) were significantly decreased. The UPLC-MS profile of the change in metabolites in T2 is listed in Table 4. A total of 4 metabolites (3-keto-4-methyzymosterol, 4-methylzymosterol, episterol and ergosterol) showed significantly different concentrations. The results reveal that the expression levels of these metabolites significantly varied among the different developmental stages. To assess metabolomic performance, we measured the end product ergosterol. The results are shown in Additional file 1: Figure S5. The m/z values and retention times of the metabolomic results are consistent with the validation measurements, indicating that the metabolomic results are reliable.

**Table 3:** Differential metabolites in ergosterol biosynthesis during T1 of *F. velutipes*

Chemical name	Fold change	log_2_(FC)	*P-*value	VIP	Significance
Squalene-2,3-epoxide	0.03114496	−5.00486	0.00020508	2.514624	Down
Isopentenyl pyrophosphate	2.35066779	1.233071	0.000103102	1.195804	Up
Dimethylallyl pyrophosphate	10.1515845	3.343633	7.55E-09	2.111362	Up
4,4-Dimethy-cholesta 8,14,24-trienol	0.31062526	−1.68675	0.001226645	1.284612	Down
4-Methylzymosterol-carboxylate	0.15222239	−2.71575	2.50E-05	1.871510	Down
4-Methylzymosterol	7.08069677	2.823891	6.65E-05	1.270038	Up
Ergosta-5,7,22,24-tetraenol	0.38993986	−1.35868	0.000401919	1.296672	Down

**Table 4:** Differential metabolites in ergosterol biosynthesis during T2 of *F. velutipes*

Chemical name	Fold change	Log_2_(FC)	*P-*value	VIP	Significance
3-Keto-4-methyzymosterol	1.56385706	0.645109	0.009914243	1.110543368	Up
4-Methylzymosterol	14.69304860	3.877062	0.000256294	2.459251368	Up
Episterol	9.165812320	3.196263	0.000112059	2.476645093	Up
Ergosterol	6.268419196	2.648102	0.000306426	2.126091340	Up

### Correlation analysis between transcripts and sterol derivatives reveals the regulatory network of ergosterol biosynthesis in *F. velutipes*

**Fig. 6:**
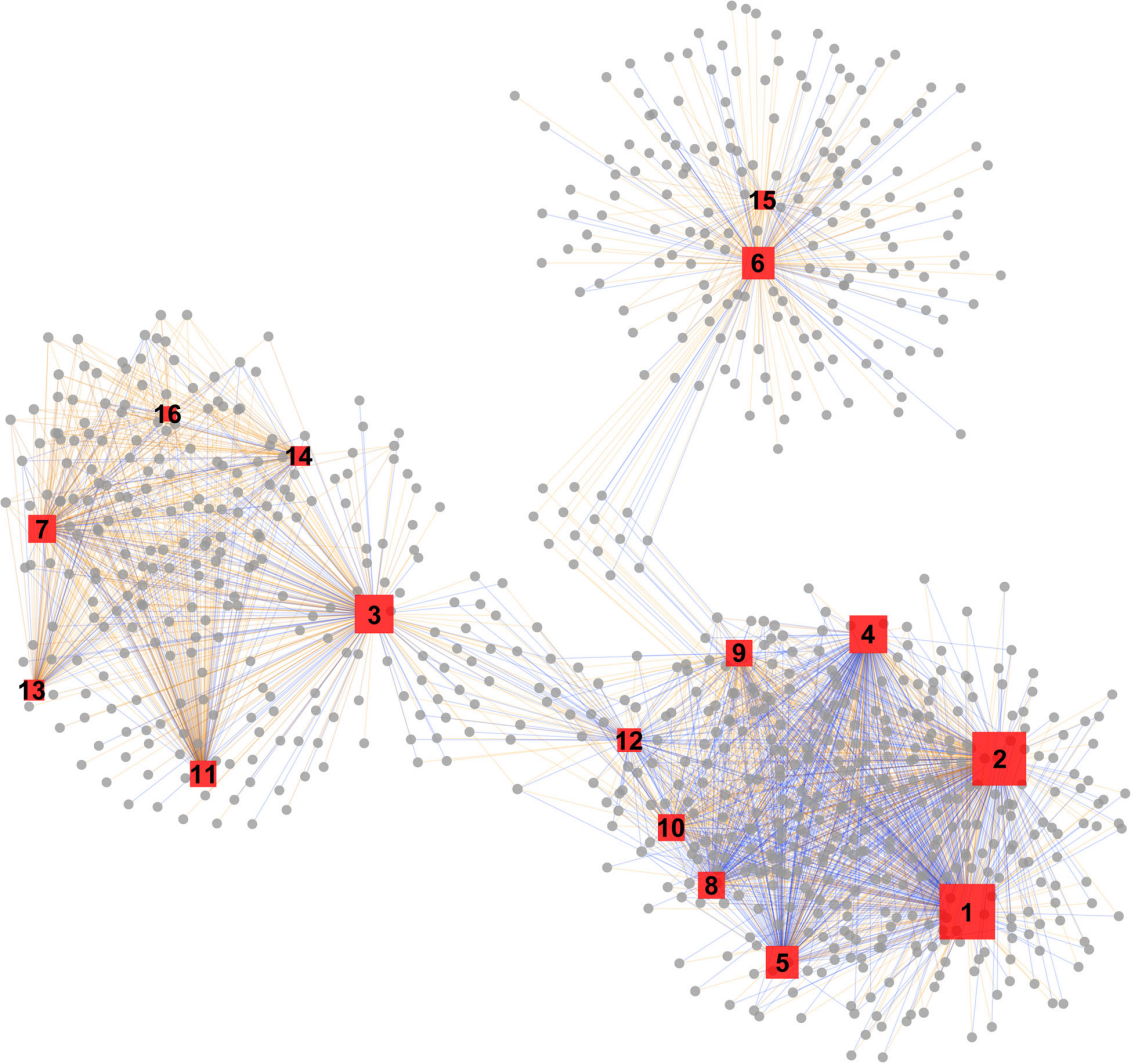
Analysis of the genome-wide connection network between regulatory genes and metabolites related to sterols at different developmental stages of *F. velutipes*. Red and gray represent metabolites and genes, respectively. The orange and blue lines represent positive and negative correlations, respectively. The size of the red area is analyzed by degree. The metabolite names represented in 1–16 are: (1) ergosta-5,7,22,24-tetraenol, (2) lanosterol, (3) mevolonate-5-phosphate, (4) fecosterol, (5) squalene-2,3-epoxide, (6) 14-demethyl lanosterol, (7) ergosta-5,7,24(28)-trienol, (8) 4-methylzymosterol-carboxylate, (9) isopentenyl pyrophosphate, (10) dimethylallyl pyrophosphate, (11) 4-methylzymosterol, (12) 4,4-dimethy-cholesta 8,14,24-trienol, (13) ergosterol, (14) farnesyl-pyrophosphate, (15) 3-keto-4-methyzymosterol and (16) episterol. The networks were visualized with Cytoscape software (version 2.8.2)

Systems biology approaches have recently emerged as highly powerful tools for discovering links between regulated genes and metabolites [30]. To unveil the underlying regulatory mechanism in sterol derivative metabolism during the development of *F. velutipes*, we performed correlation analyses of the metabolites related to ergosterol biosynthesis and the transcripts at three developmental stages of *F. velutipes*. We compared the profiles of metabolites and gene expression at three different developmental stages of *F. velutipes* using Pearson’s correlation coefficient (Additional file 1: Excel S1 and S2). The regulatory network analysis that helps us to understand the correlations between the metabolites and genes is shown in Fig. 6. The results indicated that metabolites such as ergosta-5, 7, 22, 24-butenol, lanosterol, decanoate, squalene-2, 3-epoxide and 14-desmethyllenol were more likely to be regulated in the post-squalene pathway. These results could provide insight into the relationship between the genetic control of metabolite levels and metabolic impact on gene expression.

## Discussion

In this study, a high-quality database of the *F. velutipes* transcriptome was generated based on NGS technology to illustrate the gene expression reprogramming of *F. velutipes* at different developmental stages. RT-qPCR was used to check the reliability of the transcriptomic results for *F. velutipes*. The sterol profiles of *F. velutipes* from three development stages were generated via a UPLC-Q-TOF-MS approach. We studied the changes in the expression levels of genes and metabolic content during fruiting body development and investigated regulatory networks in the fruiting process using correlation analysis. In this study, to explore the regulation of ergosterol biosynthesis, a correlation analysis was performed on metabolites and genes at three developmental stages of *F. velutipes*.

In this study, through metabonomic analysis, we found that metabolite profiles were significantly different and that the contents of ergosterol biosynthesis-related metabolites significantly changed among the three developmental stages (Tables 3 and 4). These results indicated that some of the metabolites (isopentenyl pyrophosphate and dimethylallyl pyrophosphate) present in *F. velutipes* accumulated in young fruiting bodies, while others (3-keto-4-methylzymosterol, episterol, 4-methylzymosterol and ergosterol) accumulated in mature fruiting bodies. In early fruiting body development, the accumulation of metabolites greatly contributes to the acquisition of fruiting traits [31]. In most cases, fruiting body development and metabolism are clearly interconnected and undergo major transitions that coincide with successive phases of fruiting body development [32, 33]. In addition, in this experiment, the culture media of the mycelia stage and fruiting bodies stage were two different media of PDA and sawdust, respectively. Park et al. found that the complexity of the respective culture media indicates a possible correlation between complexity and the number of expressed genes and metabolites (*F. velutipes*, PDA, MCM and sawdust) [5]. However, there is currently no clear explanation for the exceptional expression levels in *F. velutipes*.

The *ERG10* gene encodes an acetoacetyl-CoA thiolase that catalyses the formation of acetoacetyl-CoA from two acetyl-CoA molecules. When the levels of some sterols in the cell are low, the *ERG10* gene is expressed at a higher level and then regulates the activation pathway [34]. In this study, the expression level of *ERG10* was downregulated during T1, and the results indicated that the gene may be subject to feedback regulation by sterols. In previous studies, *ERG1* was identified as the key regulator of post-squalene biosynthesis in *S. cerevisiae* and *Trichoderma harzianum* [35, 36]. For example, the overexpression of *ERG1* could significantly increase ergosterol biosynthesis [37]. In *S. cerevisiae*, the deletion of *ERG26* is lethal and disrupts the synthesis of ergosterol [38, 39]. These results indicated that *ERG26* is essential for cell growth and impacts the synthesis of ergosterol. The various enzymes in the ergosterol biosynthesis pathway cooperate to tightly regulate the ergosterol content. Moreover, the genetic engineering of *F. velutipes* has been very successful [40, 41]. Genetic modification of the ergosterol pathway can be used for the production of sterols. Therefore, the study of ergosterol biosynthesis provides not only new ideas for enhancing ergosterol production but also findings applicable to the production of other economically interesting steroid molecules.

Effective genetic engineering approaches for efficient ergosterol production from the mycelia or fruiting bodies of a fungus cannot be devised until the metabolic pathway and regulation mechanism are well understood. Although the biosynthesis pathway of ergosterol in *S. cerevisiae* has been well characterized, few efforts have been made to examine ergosterol biosynthesis in *F. velutipes* [25]. The results in this paper could contribute to the improvement of the production of ergosterol and its derivatives. As shown in Fig. 6, a combined analysis of the differentially produced metabolites and genes was performed with the aim of identifying regulatory relationships. This could be a useful method for comparing the correlations of metabolites or genes between different groups [30]. A total of 13 CYPs were identified in Additional file 1: Excel S2, and the expression profiles of these genes were highly correlated with that of sterols (correlation coefficient R > 0.95). Furthermore, the majority of their expression profiles are positively correlated with sterol content profiles during development, suggesting that some of these 13 CYPs might be involved in ergosterol biosynthesis. The exact roles of these CYPs will be investigated further.

The efficiency of ergosterol biosynthesis is determined by rate-limiting enzymes, and more crucially by the optimal coordination of all enzymes [42]. The transcription factor *UPC2* has been reported to upregulate target genes involved in the biosynthesis of sterols by activating the sterol response elements in their promoter regions [43, 44]. The core motifs of the sterol-response elements have been identified in nine responsive *Candida albicans ERGs* (*ERG1*, *ERG2*, *ERG5*, *ERG6*, *ERG10*, *ERG11*, *ERG24*, *ERG26* and *ERG27*)*.* Our results revealed that the expression levels of most of these genes differed, which was consistent with the regulatory effect of *UP2C* on these genes. Most of these genes were found to be related to the post-squalene pathway, which is promising for the improvement of sterol biosynthesis in *F. velutipes* [44, 45]. The results are consistent with the results of the gene and sterol joint analysis network. However, the overexpression of specific enzymes could result in an imbalance in cellular sterol homeostasis in sterol intermediate accumulation, significantly reducing the total cell biomass yield and repressing end product formation. Therefore, it is necessary to avoid the overexpression of specific enzymes and to control the accumulation of some cytotoxic intermediates [46]. In summary, a balance between the precursor supply and the catalytic activities of enzymes should be achieved for the optimal production of ergosterol.

The in vivo biosynthesis of ergosterol is a complex metabolic process involving a variety of enzymes (at least 20), reactions and genes. Due to the different positions of each reaction in the metabolic pathway, the role of metabolic regulation is different, and therefore the effects of high expression of related genes are not the same. Some genes function well in ergosterol synthesis when properly expressed, while the overexpression of these genes may inhibit ergosterol biosynthesis. These results will shed light on the molecular mechanisms responsible for ergosterol biosynthesis in *F. velutipes*.

## Conclusions

This study explored the regulatory relationship between genes and ergosterol biosynthesis by jointly analysing transcriptional and metabolic changes at three developmental stages of *F. velutipes*. Correlation analysis highlighted regulatory genes closely associated with individual metabolites or much larger networks of genes and metabolites, thereby suggesting that a strategy based on the combined analysis of different developmental stages can be very helpful for pinpointing candidate regulatory genes linked to compositional changes and fruiting body development in *F. velutipes*. Researchers could utilize this dataset in genetic approaches to clarify the mechanism of ergosterol regulation. In summary, this study will be instrumental for further research on the biosynthesis of sterol-related metabolites in *F. velutipes*.

## Methods

### Strains and growth conditions

This article used *F. velutipes* samples from three different developmental stages, the mycelia stage (FrI), the young fruiting bodies stage (FrII) and the mature fruiting bodies stage (FrIII), as experimental materials [5, 29, 47]. T1 denotes the stage transition from mycelia to young fruiting bodies and T2 denotes the transition from young fruiting bodies to mature fruiting bodies. Mycelia, young fruiting bodies and mature fruiting bodies were acquired from Shaanxi Zhongxing Gaoke Biological Technology Co., Ltd. in Xianyang City, Shaanxi Province, China. *F. velutipes* mycelia were grown on potato dextrose agar (PDA) (20% potato extract, 2% glucose, 2% agar, pH 7.0) plates at 23–25 °C in darkness [48]. For the production of fruiting bodies, the mycelia were inoculated into 570 g of poplar sawdust, 120 g of rice bran and 65% water in a 1000 ml bottle. The cultures were incubated at 25 °C in the dark for 25~40 days and then transferred to conditions that induced fruiting. The resulting *F. velutipes* samples at three developmental stages were collected under sterile conditions, immediately frozen and stored in liquid nitrogen at − 80 °C before the metabolic content determination and RNA isolation.

### RNA extraction, library preparation and sequencing

Total RNA was extracted with the RNAprep Pure Plant Kit (Tiangen Biotech Co., Ltd., Beijing, China). The total RNA concentration, RIN value, 28S/18S and fragment size were determined using an Agilent 2100 Bioanalyser (Agilent Technologies Co. Ltd., Santa Clara, CA, USA) with the Agilent RNA 6000 Nano Kit. The purity of the samples was measured using a NanoDrop™ ultraviolet spectrophotometer. After the isolation and fragmentation of total RNA, eukaryotic mRNA was enriched by using Oligo (dT) coupled to magnetic beads. In this work, nine cDNA libraries produced from the three stages of *F. velutipes* (three replicates of each stage) were sequenced by using the Illumina HiSeq™ 4000 platform.

### Gene prediction and functional annotation of the *F. velutipes* reference genome

The reference genome of *F. velutipes* KACC42780 was downloaded from the NCBI under accession number PRJNA191921. GeneMark-ES was used to predict genes from the genome sequences [49]. The CDS sequences of each chromosome were extracted using in-house python scripts. The CDS sequences were translated using the Biopython package using the standard codon table [50]. The functions of those genes were predicted using Eggnog [51].

### Transcriptome preprocessing and differential gene expression analysis

Reads were first processed using in-house Perl scripts and filtered to remove low-quality sequences, contaminated adapters and poly-N sequences. The raw reads were mapped to the reference genome [5] using Hisat2 software [52]. HT-Seq was used to calculate the counts of each gene [53]. The Transcripts Per Kilobase Million (TPM) was calculated using the edgeR package in R programming software. Additionally, the differentially expressed genes (DEGs) were identified using the EdgeR package [54]. A gene with log_2_(fold change) ≥ 1 and a false discovery rate (FDR) < 0.01 were identified as a DEG.

### Functional annotation and enrichment analysis of DEGs

The protein sequences of DEGs were used to perform functional prediction and classification using Eggnog [51]. Functional annotation by gene ontology terms (GO) was downloaded from the UniProt (http://www.uniprot.org/uniprot) database. Kyoto Encyclopedia of Genes and Genomes (KEGG) pathway and clusters of orthologous groups (COG) annotation was performed using Blastall software against the KEGG database and the COG database, respectively, with a cut-off *E* value of 1e^− 5^. For functional enrichment analysis, GO terms of DEGs were compared to the genome background, and the corrected *p*-value less than 0.05 was set to judge the significantly enriched GO terms. The pathway enrichment analysis was performed similarly using the KEGG database, and a *p*-value less than 0.05 was the threshold.

### RT-qPCR validation

To validate internal control genes for expression analysis, the ABI StepOnePlusTM RT-PCR System (Applied Biosystems, USA) was used to perform RT-qPCR and analyse the DEGs related to ergosterol biosynthesis. Amplification primers were designed using GenScript Real-time PCR (TaqMan) Primer Design, and the primer sequences are provided in Additional file 1: Table S5. cDNA and RT-qPCR were acquired using the PrimeScript™ RT Reagent Kit and SYBR Green™ Premix Ex Taq™ П (Takara Biotech Co., Ltd., Japan). Glyceraldehyde-3-phosphate dehydrogenase (GAPDH) and β-actin were used as internal controls [55]. Each reaction was performed in a total reaction mixture volume of 20 μl containing 2 μl of first-strand cDNA as the template. The amplification program was as follows: 3 min at 95 °C followed by 40 cycles of 10 s at 95 °C and 30 s at 60 °C. Three independent technical replicates and three biological replicates for each sample were run to measure and assess the performance of RT-qPCR. The expression levels of DEGs were determined using the 2^−△△Ct^ method.

### Metabolite profiling using ultraperformance LC quadrupole time-of-flight tandem MS (UPLC-Q-TOF-MS)

In this study, 18 samples produced from *F. velutipes* at three developmental stages (six biological replicates of each stage) were tested by using UPLC-MS/MS. All samples stored at − 80 °C were placed in a freezer at − 20 °C for 30 min and then thawed at 4 °C. A 25 mg tissue sample was weighed, and placed in an EP tube, 800 μl of a cooled solution of methanol/water (1:1) was added to each EP tube, and two small steel balls were frozen and placed in each EP tube. The sample was then placed in a tissue lyser and the parameters were set to 35 Hz for 4 min. After grinding, the ball was removed and the tube was placed in a − 20 °C refrigerator for 2 h. The sample was centrifuged at 30,000×g for 15 min at 4 °C. The EP tube was carefully removed from the centrifuge, and 550 μl of each sample was transferred to a new EP tube. The sample was placed in the rack of a centrifuge, and an image was obtained according to the order of the samples on the task sheet. The samples were subsequently analysed by LC-MS, and the remaining original samples were provided to the sample manager for storage.

All samples were produced and processed following protocols for LC-MS data analysis. First, chromatographic isolation was performed by using a UPLC system (Waters, UK). Default settings were adopted for the column type, column temperature, mobile phase flow rate, mobile phase solvent ratio and other program settings [56]. The injection volume was 10 μl. The Q-TOF was run with the capillary tube and sampling voltages set at 3 kV and 40 V, respectively, in positive-ion mode and at 1 kV and 40 V in negative-ion mode. MS data were generated by Xevo G2 XS QTOF with a TOF mass ranging from 50 to 1200 Da and were scanned at 0.2 s. The MS/MS analysis was conducted to select, separate and detect precursor ions using 20 and 40 eV in different steps with a scan time of 0.2 s. When processing the data, the LE signal was received at 3 s intervals to measure the mass accuracy. A control sample was picked from every 10 samples for evaluation of the data acquisition performance of the LC-MS.

### Integrative analysis of metabolome and transcriptome data

To unveil the regulatory mechanism of ergosterol biosynthesis, we measured the associations between metabolome and transcriptome data by calculating the cosine coefficients between fold changes of the metabolite-gene pairs. For this purpose, the mean of all biological replicates from each stage in the metabolome data and the mean value of expression of each transcript in the transcriptome data were calculated. The fold changes in developmental transitions were calculated for both the metabolome and transcriptome data. Finally, the correlation matrix was imported into Cytoscape software to visualize the gene-metabolite network [57].

### Statistical analysis

We used *F* statistics and the James-Stein estimator to calculate the error variance and selected genes (fold change ≥2 and FDR < 0.01) with significant differential expression. Principal component analysis (PCA) of data from *F. velutipes* (three stages×n biological replicates) was performed to observe differences in metabolic composition and expressed genes from the three developmental stages. The gene expression data and metabolite data were standardized as Z scores. Pearson’s correlation coefficient was calculated both among the gene expression data and between the gene expression and metabolite accumulation data. For correlations with *p* < 0.01, correlation matrices were visualized using heat maps generated with MultiExperiment Viewer software version 4.0 [58]. The *p* value was calculated using an independent two-sample *t*-test.

## Supplementary information

**Additional file 1: Table S1.** The statistics of RNA-Seq data at different developmental stages of *F. velutipes*. **Figure S1.** The DEG statistics at different stages of *F. velutipes*. **Figure S2.** GO functional annotation and classification of DEGs during T1 of *F. velutipes*. **Figure S3.** GO functional annotation and classification of DEGs during T2 of *F. velutipes*. **Figure S4.** Differential expression profiles of nine DEGs at different developmental stages of *F. velutipes*. GAPDH and β-actin were used as internal controls. There were three biological replicates and three technical replicates for each gene. **Table S2.** The results of differential ions identification at different developmental stages of *F. velutipes*. **Table S3.** Identified metabolites in the ergosterol biosynthesis during T1 of *F. velutipes*. **Table S4.** Identified metabolites in the ergosterol biosynthesis during T2 of *F. velutipes*. **Figure S5.** Verification results of the end product ergosterol in metabolomics. (A) LC-MS spectrum of the ergosterol standard. (B) LC-MS spectrum of ergosterol from *F. velutipes*. Their corresponding m/z and retention time (RT) are indicated in the figure. **Excel S1.** The profiles of metabolites and gene expression at three different developmental stages of *F. velutipes* using Pearson’s correlation coefficient. **Excel S2.** Network analysis results of metabolites and genome-wide genes at three different developmental stages of *F. velutipes* by Cytoscape. **Table S5.** Primer sequences of DEGs related to ergosterol biosynthesis in *F. velutipes*.

## Data Availability

The datasets supporting the conclusions of this article are included in the article and its additional file. Raw reads data has been deposited in NCBI under accession number PRJNA592256.

## Abbreviations

COG: Clusters of orthologous groups
DEGs: Differentially expressed genes
FC: Fold change
FDR: False discovery rate
*Flammulina velutipes*: *F. velutipes*
FrI: Mycelia stage
FrII: Young fruiting bodies stage
FrIII: Mature fruiting bodies stage
FVS: *F. velutipes* sterols
GO: Gene Ontology
KEGG: Kyoto Encyclopedia of Genes and Genomes
NGS: Next generation sequencing
Q30 percentage: Percentage of bases with sequencing error rate lower than 1‰
RNA-Seq: RNA sequencing
RT-qPCR: Real-time quantitative polymerase chain reaction
SBIs: Sterol biosynthesis inhibitors
UPLC: Ultra-performance liquid chromatography
